# Whole transcriptome analysis of a reversible neurodegenerative process in *Drosophila* reveals potential neuroprotective genes

**DOI:** 10.1186/1471-2164-13-483

**Published:** 2012-09-15

**Authors:** María José Ferreiro, Naiara Rodríguez-Ezpeleta, Coralia Pérez, Michael Hackenberg, Ana María Aransay, Rosa Barrio, Rafael Cantera

**Affiliations:** 1 Developmental Neurobiology, IIBCE, Montevideo, Uruguay; 2 Genome Analysis Platform, CIC bioGUNE & CIBERehd, Derio, Spain; 3 Current affiliation: AZTI Tecnalia, Marine Research Division, Sukarrieta, Spain; 4 Functional Genomics, CIC bioGUNE, Derio, Spain; 5 Genetics Department, Granada University, Granada, Spain; 6 Zoology Department, Stockholm University, Stockholm, Sweden

**Keywords:** mRNA-Seq, *Spalt*, SALL, Neurodegeneration, Neuroprotection, *Drosophila*

## Abstract

**Background:**

Neurodegenerative diseases are progressive and irreversible and they can be initiated by mutations in specific genes. *Spalt-like* genes (*Sall*) encode transcription factors expressed in the central nervous system. In humans, *SALL* mutations are associated with hereditary syndromes characterized by mental retardation, sensorineural deafness and motoneuron problems, among others. *Drosophila sall* mutants exhibit severe neurodegeneration of the central nervous system at embryonic stage 16, which surprisingly reverts later in development at embryonic stage 17, suggesting a potential to recover from neurodegeneration. We hypothesize that this recovery is mediated by a reorganization of the transcriptome counteracting SALL lost. To identify genes associated to neurodegeneration and neuroprotection, we used mRNA-Seq to compare the transcriptome of *Drosophila sall* mutant and wild type embryos from neurodegeneration and reversal stages.

**Results:**

Neurodegeneration stage is associated with transcriptional changes in 220 genes, of which only 5% were already described as relevant for neurodegeneration. Genes related to the groups of Redox, Lifespan/Aging and Mitochondrial diseases are significantly represented at this stage. By contrast, neurodegeneration reversal stage is associated with significant changes in 480 genes, including 424 not previously associated with neuroprotection. Immune response and Salt stress are the most represented groups at this stage.

**Conclusions:**

We identify new genes associated to neurodegeneration and neuroprotection by using an mRNA-Seq approach. The strong homology between *Drosophila* and human genes raises the possibility to unveil novel genes involved in neurodegeneration and neuroprotection also in humans.

## Background

Neurodegenerative processes, which affect a high proportion of the human population worldwide, result from very complex interactions at molecular, cellular, histological and organismal levels, being progressive as well as irreversible. A great proportion of the human transcriptome is expressed in the central nervous system (CNS) under a strict spatial, temporal and quantitative control. Mutations in genes involved in a variety of molecular and cellular functions, resulting in abnormally low or high levels of their corresponding proteins, contribute to the development of some important neurodegenerative diseases such as Alzheimer or Parkinson (reviewed in [1]).

The evolutionary conservation of key mechanisms for the development, function and maintenance of the nervous tissue makes the fly *Drosophila melanogaster* a good model system for the study of human neurodegenerative diseases [1-3]. The use of this model presents various additional advantages, including the increasing number of available genetic tools to manipulate gene expression, with temporal and spatial specificity, through trans-genes encoding wild type (WT) or mutated forms of *Drosophila* or human genes [4].

The zinc finger transcription factors Spalt-like (SALL) are expressed in the CNS during embryonic development in *Drosophila* and other organisms. In humans, mutations in *SALL1* are associated with Townes Brocks Syndrome [5], while mutations in the *SALL4* gene are associated with the Duane-Radial Ray or Okihiro Syndrome [6,7]. These syndromes are characterized by limb and renal malformations, as well as nervous system defects that result in sensorineural deafness and, in some cases, mental retardation and motoneuron problems [6,8,9].

**Figure 1:**
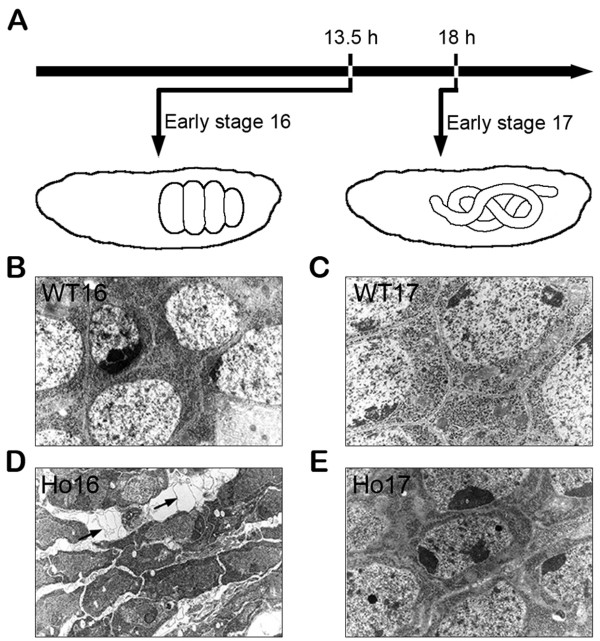
**Neurodegeneration and reversal stages of *sall *mutant embryos.** (**A**) Schematic representation of the time points and morphological features selected to identify embryos from early stage 16 (neurodegeneration stage) and early stage 17 (neurodegeneration reversal stage). (**B**-**E**) Ultrastructural comparison of the central nervous system in WT (**B**, **C**) and homozygous (Ho) *sall* mutants (**D**, **E**) at embryonic stages 16 and 17. Ho16 embryos (**D**) show smaller cell bodies, separated by enlarged extracellular space occupied by vacuoles and other membranous material (arrows), contrasting with WT16 embryos (**B**, **C**) that present normal extracellular space. This phenotype is no longer observed a few hours later in Ho17 (**E**). Panels B-D are reprinted from Cantera et al. 2002.

Two members of the SALL family are known in *Drosophilaspalt major* (*salm*) and *spalt-related* (*salr*) [10]. The transcription of *salm* starts shortly after blastoderm formation and continues throughout embryogenesis, overlapping partially with *salr*[11]. *salm* and *salr* null mutations affect several tissues, including the tracheal system [12], CNS [13] and the peripheral nervous system [14], and are embryonic lethal in homozygosis. Homozygous *sall* mutant embryos exhibit degeneration of the CNS at embryonic stage 16 (13–16 hours of development, Figure 1A, B, D). *In situ* studies of *sall* mutant embryos and *in vitro* studies of neurons generated by *sall* mutant stem cells, including time-lapse video recording, demonstrated that the mutant phenotype exhibits fragility of the nervous tissue, deficient axonal cytoskeleton and loss of cell adhesion. Consistent with these findings, a study with transmission electron microscopy showed a greatly enlarged extracellular space and several other features indicative of a degenerative process, including vacuolization and abundant membrane “whorls” and autophagosomes [13]. This suggested that *sall* controls, directly or indirectly, the transcription of genes that are important for the integrity of the CNS, possibly acting through cell adhesion and the cytoskeleton. A few hours later, at early stage 17 (16–18 hours of embryonic development, Figure 1A, C, E), part of this phenotype is reverted, suggesting a potential to recover from the neurodegenerative process. The recovery could be mediated by genetic redundancy, either as coincidental redundancy (i.e. genes performing the same function at the same developmental stage) or sequential redundancy (genes performing a similar function at different stages). In this last case, the disappearance of the ultrastructural *sall* phenotype in a few hours (from early stage 16 to early stage 17) could be partially explained by transcriptional changes taking place during this interval [13]. Genomic studies have subsequently reinforced this idea by showing that, during this time window, the *Drosophila* transcriptome undergoes rapid global changes in gene expression [15-17].

Based on the hypothesis that the recovery from neurodegeneration could be explained by a reorganization of the *sall* mutant transcriptome that partially compensates the absence of *sall* genes [13], we used mRNA high throughput sequencing (mRNA-Seq) to identify genes that might have neuroprotective function, by comparing the transcriptome of *Drosophila sall* mutant and wild type embryos from stages 16 and 17.

Our mRNA-Seq analysis demonstrates that a characteristic transcriptional signature identifies the neurodegeneration and reversal stages. Thus, the neurodegeneration phenotype observed at embryonic stage 16 in *sall* null mutants is associated with transcriptional changes in genes related to the Redox, Lifespan/Aging and Mitochondrial diseases groups, while the reversal of the neurodegeneration observed at embryonic stage 17 is associated to the regulation of genes involved in the Immune and Salt stress response groups.

## Results

### Analysis of the *sall* mutant transcriptome

The mutation *Df(2 L)32FP-5* used for the present approach is a small deficiency that covers both *salm* and *salr* genes [18]. Embryos that are homozygous mutant for these genes die at the end of embryogenesis or beginning of the first larval instar.

Total RNA was extracted from wild type (WT), *Df(2 L)32FP-5/+* (heterozygous, from now on called He) or *Df(2 L)32FP-5/Df(2 L)32FP-5* (homozygous, from now on called Ho) embryos at the two time points illustrated in Figure 1A. cDNAs from two or three independent pool-preparations for each genotype and developmental stage were sequenced. Sequencing parameters of the biological replicas of the studied genotypes are shown in Additional file 1: Table S1. DESeq package was used for measuring gene expression differences between the samples analyzed [19]. Genes showing expression differences among genotypes and developmental stages that exceeded the threshold defined as statistically significant (p <0.001) were selected for further analysis. The variance in transcript representation between differential expression (DE) results per genotype comparison is shown in Additional file 2: Table S2. Alternatively, Reads Per Kilobase of transcript per Million mapped reads (RPKMs) were also calculated [20] to corroborate the expression variability intra-samples (Additional file 3: Table S3).

**Figure 2:**
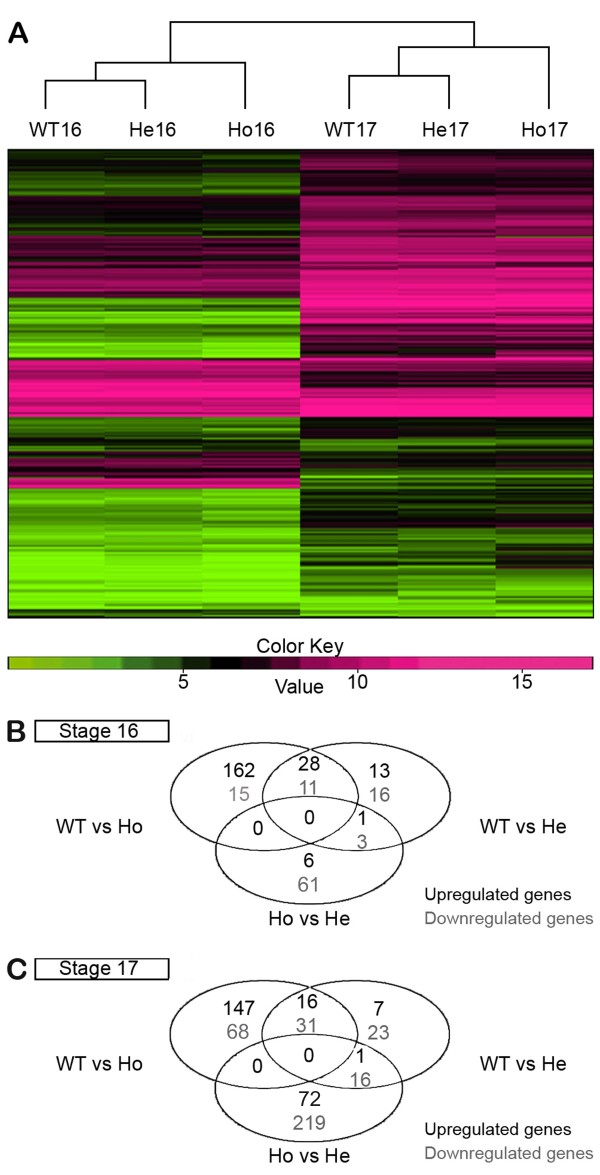
**Comparative analysis of *sall *mutant transcriptome.** (**A**) Heatmap representation of a filtered set of 2534 genes that show differential expression at p <0.001 in at least one of the following comparatives. At stages 16 or 17: WT *vs* homozygous (Ho), WT *vs* heterozygous (He) and He *vs* Ho. At the transition from 16 to 17: WT16 *vs* WT17, He16 *vs* He17, and Ho16 *vs* Ho17. Columns represent samples and rows genes. Colors represent log2 expression ratio values with pink being above and green below the row/column median level of expression (normalized gene counts) as shown by the scale (pink: genes upregulated; green: genes downregulated). (**B**-**C**) Venn diagrams of differential expression overlap between WT, He and Ho *sall* mutant embryos at stage 16 (**B**) and 17 (**C**). Numbers represent upregulated and downregulated genes (top and bottom, respectively) in the second genotype with respect to the first (e.g. WT *vs* Ho represent the number of misregulated genes in homozygous compared to WT).

We first compared the global transcriptome profile of Ho *sall* mutant embryos with those of He and WT at embryonic stages 16 and 17.We hypothesized that transcriptional misregulation associated with neurodegeneration occurs mainly at stage 16, and that genes providing neuroprotection will be regulated mainly at stage 17. The heatmap in Figure 2A shows the hierarchical clustering of all genotypes at both stages with respect to the 2534 genes that showed differential expression in at least one of the compared pairs. These results show that the transcriptome differs greatly from stage 16 to 17, as the three genotypes at each stage segregate together and separate from the other stage.

The number of genes that showed differential expression profiles in each of the comparisons between WT16, Ho16, and He16 (Figure 2B) and WT17, Ho17, and He17 (Figure 2C) is indicated by Venn diagrams. The transcriptome profiles of He and WT embryos showed differences in less than 100 genes per stage (42 genes up and 30 down at stage 16; 24 genes up and 70 down at stage 17). The Ho mutants, instead, showed misregulation of more than 200 genes, most of which were upregulated (190 genes up and 26 down at stage 16; 163 genes up and 97 down at stage 17).

The transcriptome comparison of the three genotypes at stage 16 generated a list of 249 genes that were significantly misregulated in Ho16 or He16 *sall* mutants in comparison to WT16 (Figure 2B and Additional file 4: Table S4). Out of those, we discarded 161 genes with profiles not consistent among all genotype comparisons. For instance, genes that did not show significant differences between WT16 and Ho16, nor between He16 and Ho16, but showed significant differences between WT16 and He16 were removed from the analysis. The 88 remaining genes were classified in two categories: *sall* dose-independent (47 genes) and *sall* dose-dependent (41 genes), (Additional file 4).

The transcriptome comparison of the three genotypes at stage 17 generated a list of 307 genes significantly misregulated in Ho17 or He17 *sall* mutants with regard to WT17 (Figure 2C and Additional file 4). As mentioned previously for stage 16, we discarded those genes with profiles not consistent among all genotypes comparisons (92 genes). The remaining 215 genes were classified in two categories: *sall* dose-independent (151 genes) and *sall* dose-dependent (64 genes) (Additional file 4).

We found a dosage effect of Sall on certain genes, consistent with previous results [13]. He *sall* mutant embryos have higher transcript levels than Ho for *armadillocadherin-N* and *fasciclin- 2* (Additional file 5: Figure S1A). We also found intermediate transcript values for other genes in He embryos compared to those of Ho and WT (Additional file 5: Figure S1B-C). Among these, at stage 16, we observed genes associated with tissue regeneration (CG2233, [21]), Lifespan/aging (*mtND2*, [22]; *dro5*, [23]), and salt stress (*sala*, [24]). At stage 17, some of the genes showing a dosage effect are associated with neurodegeneration and salt stress (*IM10,*[24,25]), or starvation (CG6283, [26,27]). Interestingly, in humans the Duane-Radial Ray Syndrome is caused by the deletion of one copy of *SALL4*, suggesting that also in humans two copies of a *SALL* gene are necessary to keep the correct levels of its downstream targets [28]. Furthermore, it is reported the dosage-dependent regulation of the target gene *knirps* by Sall in the *Drosophila* wing imaginal disc [29].

### Analysis of the transition between embryonic stages 16 and 17

**Figure 3:**
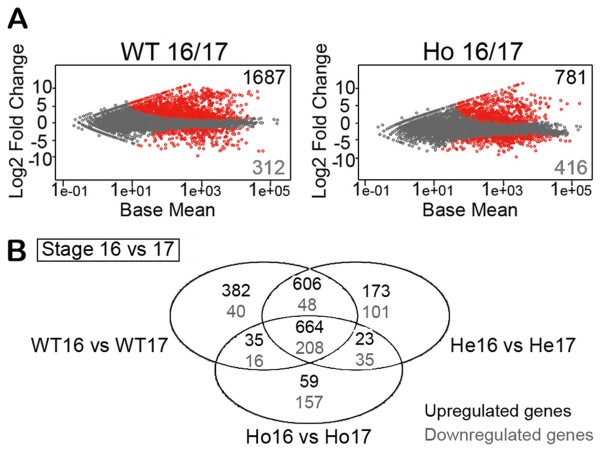
**Differential expression analysis during the transition from embryonic stage 16 to 17.** (**A**) Analysis of the transcriptome of WT and Ho *sall* mutant embryos during the transition from stage 16 to 17. Scatter plot of log2 fold gene expression changes from the first to the second condition *versus* the mean counts for each compared pair. Unique counts normalized by the effective library size were used. Each point corresponds to a gene. Genes without significant change in their expression levels appear along the zero value of the y-axis (grey), and those whose transcription exceeds the threshold defined as statistically significant (p < 0.001, differentially expressed at 0.1% False Discovery Rate) are shown in red, above the y-axis zero value (genes upregulated at the transition from stage 16 to 17, number in black) or below (genes downregulated at this stage transition, number in grey). (**B**) Venn diagrams of differential expression overlap during the transition 16 to 17 of all genotypes analyzed. Genotypes analyzed: WT16 *vs* WT17, He16 *vs* He17, and Ho16 *vs* Ho17. Numbers indicate upregulated and downregulated genes for each comparison (top and bottom numbers, respectively) at stage 17 with respect to 16.

During the transition from early stage 16 to early stage 17, the number of genes that change expression differed between WT and Ho *sall* mutant embryos (Figure 3). In WT embryos, this transition comprised the upregulation of 1687 genes and downregulation of 312 genes (Figure 3A). A similar change was detected in He *sall* mutant embryos (data not shown). However, Ho embryos showed less upregulated genes and more downregulated genes compared to WT (781 genes upregulated and 416 genes downregulated; Figure 3A). Despite these differences, Ho *sall* mutant embryos are able to pass to stage 17 (tubular intestine, Figure1A), and die shortly after. Therefore, the genes necessary for the transition from stages 16 to 17 are likely included in the 872 genes that change during the transition in the three genotypes (664 upregulated and 208 downregulated; Figure 3B).

### modENCODE comparison with differential expression results

**Figure 4:**
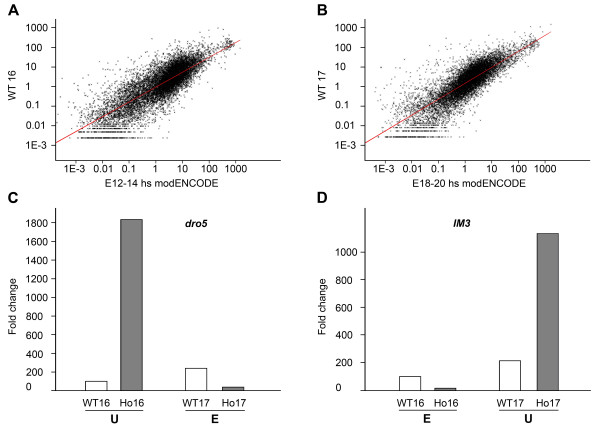
**Validation of data from mRNA-Seq.** (**A**, **B**) Graphical representation of the correlation between modENCODE expression data of E12-14hs embryos and the mRNA-Seq expression data for WT16 (**A**) and E18-20hs embryos with the mRNA-Seq expression data for WT17 (**B**). Correlation coefficients are 0.79776 and 0.81202, respectively. (**C**, **D**) Graphical representation of the expression of *dro5* (**C**) and *IM3* (**D**) obtained by Q-PCR in the indicated genotypes. Ho: homozygous *sall* mutant embryos. U (upregulated) and E (equal) refer to the expression analysis by mRNA-Seq of these genes at the indicated genotypes’ comparisons.

We compared our data with transcriptome data published by the modENCODE project [17]. We found good correlation (Pearson product–moment correlation coefficient) between our mRNA-Seq data and that of modENCODE, comparing our WT16 data with E12-14hs data (r = 0.79776, Figure 4A) and our WT17 data with E18-20hs (r = 0.81202, Figure 4B). 1175 genes are shared among the upregulated (64.35%) and 412 genes among the downregulated (39.16%) in both datasets.

### Validation of differential expression results

In order to validate our results, we performed Q-PCR for selected genes that presented different number of reads per genotype and stage (Additional file 6: Figure S2). We chose two genes belonging to the Immune response group that showed different profiles in the mutant. According to our sequencing results, *dro5* was highly upregulated in Ho16, but not in Ho17. These differences were confirmed by Q-PCR (Figure 4C). Conversely, according to our mRNA-Seq results, *IM3* did not show significantly different expression levels between Ho16 and WT16, but was highly upregulated in the mutants at stage 17. The same results were obtained by Q-PCR (Figure 4D). Interestingly, we noted that *IM3* and *dro5* genes show Sall binding sites in their promoters, and that those sites are conserved among several *Drosophila* species (Additional file 7: Figure S3).

### Identification of genes potentially involved in neurodegeneration

The transcriptome comparison of WT and homozygous *sall* mutant embryos generated a list of 620 genes significantly misregulated at stages 16 and/or 17, including those that changed during the transition 16 to 17 exclusively in the homozygous embryos (Additional file 8: Table S5). The Additional file 9 shows the complete list of Gene Ontology (GO) categories enriched among these 620 genes, after a non-biased analysis with VLAD online tool ( http://proto.informatics.jax.org/prototypes/vlad-1.0.3). This analysis revealed an enrichment of genes involved in the oxidation-reduction process in the WT16 vs Ho16 comparison, as well as an enrichment of serine-type endopeptidases in the WT17 vs Ho17 and the Ho16 vs Ho17 comparisons. Next, we performed a biased GO analysis of these 620 genes, using functional categories selected after an extensive literature search (Additional file 10: Tables S6, S8 and S10). Through our bibliographic search we were able to identify the involvement of some of these genes in neurodegeneration or neuroprotection, either in *Drosophila* or in other organisms (72 genes highlighted in red in Additional file 8). Our hypothesis assumes that changes in transcript levels in Ho16 in comparison to WT16 (Figure 2B) might reveal genes contributing directly or indirectly to the neurodegenerative phenotype as well, as other genes with several other functions not relevant for this study.

**Figure 5:**
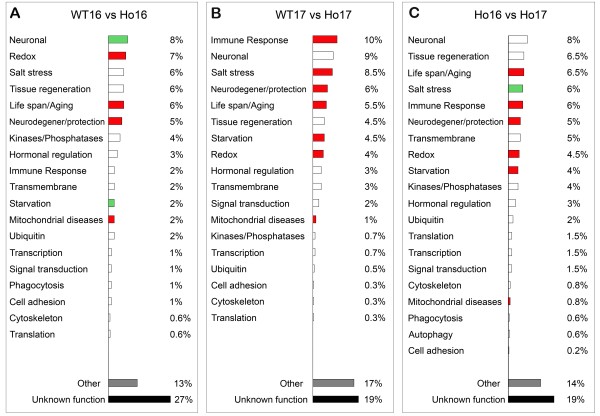
**Classification of genes misregulated in homozygous *sall *mutant embryos in Functional Categories.** (**A**-**C**) Graphic representation of the main functional groups enriched in misregulated genes in homozygous *sall* mutants (Ho), expressed as percentage of genes in each group. In red are marked the groups significantly overrepresented with respect to the total *Drosophila* genome with p < 0.01 and in green with p < 0.05. (**A**) Classification of the genes misregulated in Ho16 compared with WT16. (**B**) Classification of the genes misregulated in Ho17 compared to WT17. (**C**) Classification of the genes misregulated in Ho embryos at the transition from stage 16 to 17.

Classification of all the genes misregulated in Ho16 with respect to WT16 in Functional Groups revealed that 8% belong to the Neuronal group, 7% to the Redox group, followed by Salt stress (6%), Tissue regeneration (6%), Lifespan/Aging (6%), and the Neurodegeneration/Neuroprotection groups (5%), (Figure 5A and Additional file 10: Table S6), and by other groups with less than 5% representation. Gene Ontology (GO) analysis of these genes showed that the groups associated with Neurodegeneration/Neuroprotection, Redox, Lifespan/Aging and Mitochondrial diseases were significantly represented (p < 0.01) among Ho16 misregulated genes, with respect to their representation in the whole *Drosophila* genome (Figure 5A, highlighted in red). Neuronal and Starvation groups were also significantly represented (p < 0.05; Figure 5A, highlighted in green). Interestingly, 27% of genes misregulated in Ho16 embryos with respect to WT16 have no biological function experimentally assigned or suggested yet, and could be considered as new genes potentially associated to neurodegeneration.

Classification of genes that change transcript levels in Ho16 when compared to He16 in Functional Groups revealed that 8% belong to the Salt stress group, 7.5% to the Neuronal group, followed by Tissue regeneration (6%), Mitochondrial diseases (6%), Lifespan/Aging (6%), and Redox (5%), and by other groups with less than 5% representation (Additional file 10: Table S7 and Additional file 11: Figure S4A). GO analysis of these genes showed that the Redox group was significantly represented (p < 0.01) among Ho16 misregulated genes, with respect to their representation in the whole *Drosophila* genome (Additional file 11: Figure S4A, highlighted in red). Neuronal, Mitochondrial diseases, Neurodegeneration/Neuroprotection, Hormonal regulation and Starvation groups were also significantly represented (p < 0.05; Additional file 11: Figure S4A, highlighted in green). These results are coincident with those showed in the WT16 vs Ho16 comparative (Figure 5A).

### Identification of genes potentially involved in neuroprotection

Our hypothesis also assumes that among the genes with significantly altered transcript levels in Ho17 in comparison to WT17 (Figure 2C) might be those with neuroprotective function.

Classification of all these genes in Functional Groups showed that 10% of them belong to the Immune response group, 9% to the Neuronal group, followed by Salt stress (8.5%), Neurodegeneration/Neuroprotection (6%), Lifespan/Aging (5.5%), and other functional groups (each representing less than 5% of the misregulated genes), (Figure 5B and Additional file 10: Table S8). GO analysis of these genes showed that Immune response, Salt stress, Neurodegeneration/Neuroprotection, Lifespan/Aging, Starvation, Redox and Mitochondrial diseases groups were significantly overrepresented (p < 0.01) among Ho17 misregulated genes, with respect to their representation in the whole *Drosophila* genome (Figure 5B, highlighted in red). The Neuronal group was also significantly overrepresented (p < 0.05; Figure 5B, highlighted in green). Interestingly, 19% of genes misregulated in Ho17 with respect to WT17 have no biological function experimentally assigned or suggested yet, and could be potentially associated to neuroprotection.

We also compared gene transcript levels in Ho17 with respect to He17 (Additional file 10: Table S9 and Additional file 11: Figure S4B). Classification of these genes in Functional Groups revealed that 10% belong to Salt stress and 10% to Immune response, followed by Mitochondrial diseases (7%), Neuronal (6%) and Lifespan/Aging (5%), and by other groups with less than 5% representation. GO analysis of these genes showed that Salt stress, Immune response, Mitochondrial diseases, Neurodegeneration/Neuroprotection and Starvation groups were significantly represented (p < 0.01) among Ho17 misregulated genes, with respect to their representation in the whole *Drosophila* genome (Additional file 11: Figure S4B, highlighted in red). These results are coincident with those showed in Figure 5B. The homozygous embryos show overrepresentation of genes belonging to the same groups than the comparison between WT17 vs Ho17, supporting the concept that heterozygous embryos are more closely related to WT than to homozygotes.

Finally, our hypothesis assumes that genes with neuroprotective function might also be found among the genes with significantly altered transcript levels at the transition from stage 16 to 17 only in homozygous *sall* mutant embryos but not in WT or heterozygous (Figure 3B). Classification of all these genes in Functional Groups showed that 8% belong to the Neuronal group, 6.5% to Tissue regeneration, 6.5% to Lifespan/Aging, 6% to Salt stress, 6% to Immune response, 5% to Neurodegeneration/Neuroprotection and 5% to Transmembrane groups, followed by other groups with less than 5% representation (Figure 5C and Additional file 10: Table S10). GO analysis of these genes showed that the groups associated with Lifespan/Aging, Immune response, Neurodegeneration/Neuroprotection, Redox, Starvation and Mitochondrial diseases were significantly overrepresented (p < 0.01) with respect to their incidence in the whole *Drosophila* genome (Figure 5C, highlighted in red). The Salt stress group was also significantly overrepresented (p < 0.05; Figure 5C, highlighted in green). The Neurodegeneration/Neuroprotection and Redox groups were significantly overrepresented among downregulated genes (p = 2.15 × 10^-09^ and p = 1.29 × 10^-04^, respectively), while the groups for Immune response, Starvation and Mitochondrial diseases were significantly overrepresented among upregulated genes (p = 8.77 × 10^-12^, p = 2.75 × 10^-12^ and p = 1.36 × 10^-05^, respectively). Finally, 19% of genes misregulated in homozygous embryos at the transition between stages 16 and 17 have no biological function experimentally assigned or suggested yet, and could be considered as new genes potentially associated to neuroprotection.

When we analyzed the transcriptional changes in the 16 to 17 transition in WT embryos, we saw that only the Immune response, Starvation, Tissue regeneration and Mitochondrial diseases groups are overrepresented with respect to the whole genome (Additional file 10: Table S11 and Additional file 11: Figure S4C). However, this overrepresentation is in a lower proportion than in Ho16 vs Ho17 comparison, which is in agreement with our hypothesis that the transcriptional changes that we see in the 16 to 17 transition in homozygous embryos are specifically developed by the homozygous mutant embryos to try to compensate their neurodegeneration at stage 16.

## Discussion

### Analysis of the transition between embryonic stages 16 and 17

Several waves of coordinated down- or upregulation in the expression of large gene clusters have been detected throughout the life cycle of *Drosophila* using microarrays technology [15,16,30]. A major transcriptional shift occurs between 11 and 18 hours of embryonic development [16], which partially overlaps with the time interval studied here. Our study confirms that the *Drosophila* transcriptome changes quickly at the transition between stage 16 and 17 of embryonic development, within less than 4–5 hours, and shows that a major shift occurs also in *sall* heterozygous and homozygous mutants, although there are clear differences in the transcriptome of each genotype. The 872 genes that we found to change transcript levels from stage 16 to 17 in all three genotypes studied here, most probably include all or most of the genes that are truly necessary for this developmental transition.

### Analysis of the *sall* mutant transcriptome

The transcriptome of homozygous *sall* mutant embryos was clearly different from those of heterozygous and WT embryos at both stages. Most of the genes misregulated in homozygous mutants were upregulated at both stages, in agreement with studies showing that Sall proteins act as transcriptional repressors in *Drosophila* and vertebrates [31-33]. The finding that the majority of misregulated genes in embryos with a neurodegenerative phenotype was upregulated adheres to a general trend established by genomic studies of neurodegenerative processes in *Drosophila* CNS tissue, for example, in *parkin* mutants [34] and in flies with neurodegeneration caused by transgenic expression of *alpha-synuclein* or mutated *tau*[25]. The opposite relationship was found in flies with retinal degeneration caused by poly-glutamine expression [35].

### Identification of genes potentially involved in neurodegeneration

In a previous study we described in detail the phenotype of *sall* mutant embryos, which includes several landmarks of degeneration, such as fragility of the nervous tissue, deficient axonal cytoskeleton, loss of cell adhesion, enlarged extracellular space, vacuolization and abundant membrane “whorls” and autophagosomes [13]. Other embryonic phenotypes have been described, including malformation of the tracheal [12] or the peripheral nervous system [14]. Even if some aspects of the phenotype could be attributed to reasons different than neurodegeneration, the prominent phenotype shown in the CNS prompted us to assume that genes significantly up or down-regulated at stages 16 and/or 17, and during the transition 16 to 17 specifically in the homozygous *sall* mutant embryos might include many of the genes associated with the neurodegenerative phenotype and its reversal.

Classification of all the genes whose transcription was misregulated in Ho16 with respect to WT16 in Functional Groups, revealed that only 5% of them have been previously associated to neurodegeneration. The overrepresentation of the Neurodegeneration and the Redox groups among Ho16 misregulated genes (p = 1.28 × 10^-07^ and p = 7.59 × 10^-07^, respectively) is in accordance with previous works about neurodegeneration. The misregulated genes belonging to the Redox group include *sugarless* (*sgl),* associated with the Wingless pathway and to resistance to oxidative damage [36,37]*; l(2)01289*, which protects from beta-amyloid toxicity [38]; nine genes codifying for cytochromes (*mt: Cyt-b, mt: CoI-III, Cyp4g1, Cyp4p2, Cyp6a23, Cyp12c1, Cyp304a1*) and six genes codifying for NADH-ubiquinone oxidoreductase chains (*mt:ND1-ND6*). A predominance of the Redox group was also found in flies with neurodegeneration caused by mutation in *parkin*[34], expression of human beta-amyloid [38] or hyperoxia [39]. The Mitochondrial disease group was also strongly represented among Ho16 downregulated genes (p = 5.25 × 10^-03^) and included among others *technical knockout*, a model for mitochondrial disease [40]. As a whole, these results reinforced the abundant data supporting the existence of a strong correlation between redox imbalance and neurodegeneration [41-45] and strongly indicate that SALL proteins are important for normal mitochondrial function. Interestingly, genes encoding mitochondrial proteins represent the major group of genes that are misregulated both, in our model, and in muscle tissue where *salm* expression was silenced [46] (Additional file 12: Table S12).

Gene Ontology analysis of all the genes misregulated in Ho16 with respect to WT16 showed that the groups Neuronal (genes associated with nervous system development and function) and Starvation were also significantly represented (p < 0.005). Some examples are *Ama*, which encodes a protein important for neuronal cell adhesion (ligand of Neurotactin) also misregulated by polyglutamine expression [35]; *Hsp23* and *Hsp26*, that encode heat shock proteins expressed in CNS and other tissues, previously associated with longevity [47], mitochondrial diseases [40] and tissue regeneration [21]; *nicotinic Acetylcholine Receptor α 34E* (*nAcRα-34E*), that is expressed in the CNS of *Drosophila* larva and adult, encodes a protein with nicotinic acetylcholine-activated cation-selective channel activity; and *CG4306*, identified as a *Drosophila* Orb2 target gene, involved in neuronal growth, synapse formation, and protein turnover [48].

### Identification of genes potentially involved in neuroprotection

The classification in Functional Groups of all the genes misregulated in Ho17 with respect to WT17 and in Ho17 with respect to Ho16, allowed us to identify genes potentially involved in the reversal of neurodegeneration. We found that 5% of these genes have already been associated with Neurodegeneration/Neuroprotection. *Hsp70Bc* is a paradigmatic example, with ample documentation of a neuroprotective function in flies with neurodegenerative phenotypes caused by *parkin* mutations or other reasons (reviewed by [39,49,50]*.* We propose that many of the remaining Ho17 misregulated genes (95%) are probably also relevant for neuroprotection.

The Immune group was particularly overrepresented among the genes upregulated in Ho17 (p = 6.48 × 10^-21^). Among others, we found upregulation of *Tsf1* and seven IM genes (Immune Induced Molecules: *IM1-4, IM10, IM14, IM23*) and downregulation of *Ect3.* Several of these genes (*IM1, IM4, Tsf1*) are regulated during *Drosophila* tissue regeneration [21]. We noticed that during normal development many of these genes show a sharp, very large peak of expression during metamorphosis [17] a time when the nervous system is radically remodeled. The importance of the Immune response group was previously demonstrated in other genomic studies of *Drosophila* neurodegeneration [25,34,38,39]. *IM4* is the second most upregulated gene in *parkin* mutants [34], *IM10* is upregulated in flies with neurodegeneration caused by mutated human *tau*[25], and *Ect3* is the homolog of *GLB1*, a human gene associated with mental retardation [51]. Other misregulated genes belonging to the Immune response group are several members of the Jonah gene family (*Jon25Bi-iii, Jon44E, Jon65Ai* and *Jon99F*), which encode proteases and have a clear involvement in proteolysis. Two of them (*Jon25Bi* and *Jon25Bii*) are also associated with mitochondrial function [40].

It has been suggested that the upregulation of immune genes in *Drosophila parkin* mutants might be related with inflammation and other immunological reactions seen in the nervous tissue of patients with neurodegenerative diseases. However, a note of caution is warranted. Individual genes can have several functions and those annotated within the immune group in *Drosophila* are not an exception. The kappaB transcription factors encoded by *dorsaldif* and *relish*, for example, besides being regulators of the immune response to pathogens, are also key players in pathways that govern the development of the body axis, as well as the development and differentiation of blood, muscle and neuronal cells [52,53]. We believe that beyond the possible engagement of immune genes in broad stress responses, including those constitutive to neurodegenerative processes, some might have neuroprotective functions as suggested here by their upregulation during the reversal of the phenotype. Future studies should address these possibilities, taking advantage of the abundance of mutants and genetic tools available for this purpose in *Drosophila*.

In addition to the Immune response group, the Neurodegeneration/Neuroprotection group is also highly represented among Ho17 misregulated genes (p = 1.05 × 10^-12^). Besides Hsp70Bc, named above, other examples of this group are *Cyp6a8, CG2065, CG11825, GstE1Prx2540-1* and *Prx2540-2* that are associated to common neurodegenerative diseases, such as Parkinson and Alzheimer [25,34,38,39,54]. *CG16727Prx2540-1* and *Prx2540*-2 are additionally associated with the Mitochondrial diseases group [40], that is also well represented among Ho17 misregulated genes (p = 0.00165). We found some genes implicated in mental retardation, like *LKR*[51], also implicated in the stress response to starvation (Starvation group). This last group is highly represented as well among Ho17 misregulated genes (p = 8.36 × 10^-15^), by genes like *Osi6CG10814, GRHR, ACC, CG18135, CG10918, CG9757, Prx2540-1* and *Prx25402*[26,55-57]. *Edg91GstE1* and *caz* are examples of genes misregulated in Ho17, also altered during tissue regeneration [21], while *Cyp6a13, Nplp4, mthl8, Prx2540-1* and *Prx2540*-2 are other examples of Ho17 misregulated genes that have been associated with Lifespan/Aging group (p = 1.96 × 10^-6^; [58-61]).

### A salt-stress response might be activated during the reversal of neurodegeneration

An interesting observation is that a substantial proportion (between 6 and 8.5%) of the genes misregulated in Ho16 and Ho17 or during the transition between both stages was also misregulated in response to osmotic stress caused in WT flies by dietary administration of excessive salt [24]. This proportion is statistically significant with respect to the whole *Drosophila* genome among Ho17 upregulated genes (p = 1 × 10^-8^). The abundant representation of genes upregulated during the response to salt stress [23] in our mutant samples and, in particular, their enrichment in homozygous embryos by stage 17, suggests that a salt-stress response might be activated during the reversal of the neurodegenerative phenotype. This correlation could explain the phenotype observed by electron microscopy in the brain of Ho16 embryos ( [13]; Figure 1), because the extraordinary enlargement of the extracellular space, shrunken neuronal cell bodies and smaller axonal diameter observed at this stage, could represent an osmotic imbalance driving water from the cells. Later on, at stage 17 (neurodegeneration reversal), the upregulation of genes from the salt stress response could be part of the mechanism mediating the disappearance of this phenotype. If this hypothesis is correct, SALL proteins are dispensable for mounting a rapid response to a hyperosmotic condition, despite the fact that *salm* was one of the genes with highest upregulation 4 hours after the response to salt stress was initiated [24]. The correlation between *salm* and osmotic regulation is nevertheless intriguing, as mutations in human and mouse *SALL* genes have been firmly associated with severe pathologies of kidney development and function (reviewed in [10]).

## Conclusions

**Figure 6:**
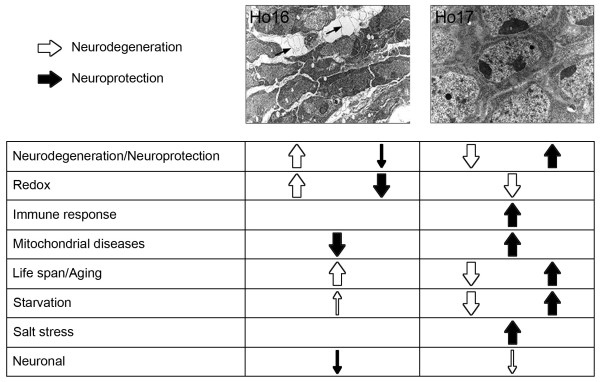
**A transcriptional signature identifies the stages of neurodegeneration or neuroprotection in homozygous *sall *mutant embryos.** (**A**) The groups of genes significantly over- or underrepresented in each stage are indicated with arrows oriented up- or downwards, respectively. Black arrows indicate genes putatively associated with neuroprotection, while white arrows represent those groups putatively associated with neurodegeneration. Big arrows symbolize groups of genes significantly represented with p < 0.001 and small arrows groups significantly represented with p < 0.05. Neurodegeneration of homozygous *sall* mutants at stage 16 coincides with the upregulation of genes associated principally to the groups of Neurodegeneration, Redox, Lifespan/Aging and Starvation, and downregulation of other genes belonging to the Redox group, and genes from Mitochondrial diseases, Neuronal and Neuroprotection groups. The recovery of the neurodegenerative phenotype at stage 17 coincides with the upregulation of genes of the Immune response group, as well as genes from Mitochondrial diseases and Salt stress groups, and some from Lifespan/Aging, Starvation, and Neurodegeneration/Neuroprotection groups. This neurodegeneration reversal stage coincides also with the downregulation of genes belonging to the Redox group, and of other genes belonging to Neurodegeneration, Lifespan/Aging, Starvation and Neuronal groups. The two photos are reprinted from Cantera et al., 2002.

The main contribution of this work is the identification of a set of genes with abnormally high or low number of transcripts during the reversal of a neurodegenerative phenotype (Figure 6), most of which could be newly associated either to neurodegeneration or neuroprotection. To our knowledge, this is the first study that employs mRNA-Seq to approach this issue, comparing the transcriptome of *Drosophila* embryonic CNS with a temporal resolution of less than 5 hours (stages 16 and 17 of embryonic development).

Our analysis included not only the transcriptomic comparison of homozygous *sall* mutant and WT embryos at neurodegeneration and reversal stages, but also with heterozygous *sall* mutants, which do not develop the neurodegenerative phenotype observed in the homozygous. This allowed us to better identified genes that could contribute to neuroprotection.

Interestingly, several of the identified genes at stages corresponding to neurodegeneration and its reversal are not yet functionally annotated and could be experimentally associated to neurodegeneration and/or neuroprotection in future studies. Our analysis also suggests for the first time, an involvement of *Drosophila sall* genes in maintaining or regulating normal mitochondrial function. In addition, our results suggest that a salt-stress response might be activated during the reversal of the neurodegenerative phenotype. This could be a general phenomenon mediating neuroprotection not only in our model of study, but also in other neurodegeneration processes, that should be addressed in further experiments.

Given the strong homology exhibited by *Drosophila* and human genes, our study might help to better understand human neurodegenerative diseases by unveiling novel genes involved in both neurodegeneration and neuroprotection.

## Methods

### Selection of embryos and RNA extraction

*Df(2 L)32FP-5* corresponds to a small deficiency on chromosome 2 that lacks both *salm* and *salr* genes [31]. As *Df(2 L)32FP-5* stock is homozygous lethal in late embryonic stages, this deficiency is maintained as an heterozygote stock with a *CyO*-GFP balancer (*Df(2 L)32FP-5/CyO-GFP*), which also facilitates embryo selection by GFP expression. The mutant stock was back-crossed with the balancer stock until all the chromosomes, except the one carrying the mutation, were substituted before the selection of the embryos.

Embryos were collected at early stage 16 (13–14 hours of egg development at 25°C) and early stage 17 (around 16 hours) based on midgut morphology and GFP expression (Figure 1A). WT embryos were selected from an *Oregon R* fly stock. Homozygous *Df(2 L)32FP-5/Df(2 L)32FP-5* embryos were selected from a *Df(2 L)32FP-5/CyO-GFP* fly stock by the absence of GFP. Heterozygous *Df(2 L)32FP-5/+* embryos were also selected by the absence of GFP from the F1 progeny of *Df(2 L)32FP-5/CyO-GFP* females crossed with *white*- males. Fifty embryos per tube were collected in 20 μl of RNAlater (Ambion) and frozen in liquid nitrogen. Between two and three different pools (50 embryos each) were chosen per genotype and stage to obtain biological replicates for each condition.

Total RNA extracts were obtained from all samples, using the “Cells to cDNA II” kit (Ambion) according to the manufacturer’s instructions, quantified using Nanodrop (Thermo Scientific) and quality assessed by Agilent 2100 Bioanalyzer (Agilent).

### Expression analysis by PCR

Mutant samples were confirmed by PCR analysis of *sall* expression. Selected genes were validated by Q-PCR in independent RNA samples per genotype. RNA samples were first treated with 2 μl of DNaseI for 15 minutes at 37°C followed by enzyme inactivation for 5 minutes at 75°C. Two micrograms of RNA were retrotranscribed with random primers using the *SuperScript III First-Strand Synthesis System* (Invitrogen) at a 100 μl volume per reaction, following manufacturer’s instructions. Specific oligonucleotides for *salmdro5IM3* and *RpL32* were designed using the primer blast tool from NCBI website [62] (Additional file 13: Table S13). *RpL32* was used as a control.

For Q-PCR, EvaGreen master mix (Biotium) and mi-Hot *Taq* mix (Metabion) were used. Reactions were performed in 20 μl reaction volume (20 ng of cDNA, 1x mi-Hot *Taq* mix, 1x Eva Green mix, 0.1 μM of each primer Forward and Reverse) in a CFX-thermocycler (BioRad) as follows: 95°C for 10 min, 40 cycles of 95°C for 15 seconds and 60°C for 1 min, and a final extension of 95°C for 1 min. A melt curve from 60 to 95°C, with 0.5°C temperature increment every 5 seconds, was carried out. All the reactions were run in agarose gels stained with ethidium bromide for validation of amplification specificity. *RpL32* was used as a control.

### RNA sequencing, annotation and differential expression

cDNAs from two or three independent pool-preparations per genotype and developmental stage were sequenced. mRNA-Seq libraries were obtained out of 1 μg of total RNA per pool following the mRNA Sequencing Sample Preparation kit’s instructions (Illumina Inc.). In summary, poly-A containing mRNA molecules were isolated using poly-T oligo-attached magnetic beads; then, a random chemical fragmentation of the mRNA was followed by cDNA synthesis, ligation of Illumina’s sequencing universal adaptors and PCR amplification of ligated fragments. Libraries containing inserts between 80 and 120 bp (libraries sizes ranging from 200 to 250 bp) were quantified by Q-PCR, clustered and amplified on flow-cell lanes and sequenced in a Genome Analyzer II (Illumina Inc.) for 36 or 38 cycles. The sequences were submitted to NCBI Sequence Read Archive (SRA) ( http://trace.ncbi.nlm.nih.gov/Traces/sra/sra.cgi?view=studies) under submission ID: SRA048981.1 and to Gene Expression Omnibus (GEO) database under accession number GSE38664.

Short reads were aligned to the *Drosophila melanogaster* genome version FlyBase r5.22 with Bowtie [63] allowing using default options, but selecting only the best alignment (−−best). Reads were annotated with the R/Bioconductor Genominator package and differential expression measured with the DESeq package [19]. Aligned reads were also normalized by RPKMs [20].

### Gene Ontology and other bioinformatics analysis

Preliminary non-biased gene ontology (GO) analysis was carried out using VLAD online tool [64]. Then, functional groups lists of genes were manually elaborated based on the literature and the following databases: VLAD [64], AMIGO [65], GATHER [66], GOTM [67] and FlyBase [68]. Homozygous *sall* mutant misregulated genes found in our analysis (at stage 16, 17 and at the transition between both stages), were then classified in our “Functional Groups reference lists”. The following functional groups were constructed from specific literature sources: Tissue regeneration [21]; Salt stress (genes overexpressed 4hs after salt treatment, [24]); Mitochondrial diseases [40]; Lifespan/Aging [59,60,69-71]. Genes belonging to functional groups different from selected “Reference Groups” were categorized under the generic term “Other”. Genes with no function experimentally demonstrated or suggested yet, were classified as “Unknown function”. We then calculated and graphically represented the percentage of *sall* mutant misregulated genes at stage 16 (WT16 vs Ho16; He16 vsHo16), 17 (WT17 vs Ho17; He17 vs Ho17) and at the transition between both stages (Ho16 vs Ho17; WT16 vs WT17) belonging to each “Functional Group reference list”. Finally, we performed Gene Ontology (GO) analysis using the FatiGO software of the BABELOMICS package [72]. Additional file 14: Table S14 shows the genes in each Functional Group category used in this study.

For the analysis of Sall binding sites we used the MatScan software [73], demanding at most 1 substitution from the published consensus [11] and using the BDGP R5/dm3 genome assembly as reference.

We compared our sequencing data with those from Graveley et al. [17] in two different ways: 1) We calculated Pearson correlation coefficients between the modENCODE RNA-seq data from 12 to 14 h embryos (SRX015647) and our WT16 data and between the modENCODE RNA-seq data from 18 to 20 h embryos (SRX015650) and WT17; and 2) we determined the number of shared genes in the WT 16/17 and 12–14 h/18–20 h transitions, both for up and down-regulated. For both analyses we carried out first a median-normalization of the data in order to transform the expression values into a comparable scale. In the second analysis we consider a gene to be differentially expressed if it is at least 4-fold over- or under-expressed.

For the comparison of our data with that from the silencing of *salm* in the muscle, we compared the homozygous *sall* mutant misregulated genes found in our experiment with the top 500 genes of the microarrays of *salm* downregulated pupal muscle [46].

## Abbreviations

CNS: Central Nervous System; GFP: Green Fluorescent Protein; GO: Gene Ontology; He: Heterozygotes; Ho: Homozygotes; mRNAseq: mRNA sequencing; Q-PCR: Quantitative Polimerase Chain Reaction; RPKM: Reads Per Kilobase of transcript per Million mapped reads; *Salm*: *Spalt major*; *Salr*: *Spalt related*; WT: Wild type.

## Competing interests

The authors declare that they have no competing interests.

## Authors’ contributions

MJF designed and performed all the molecular genetic experiments, participated in the interpretation of the results and in the writing of the manuscript. NR participated in the bioinformatic analyses and in the interpretation of the results. CP provided the fly stocks. MH participated in the bioinformatic analyses. AA coordinated and performed the mRNA sequencing and participated in the interpretation of the results and the bioinformatic analyses. RB and RC conceived, designed and coordinated the study, participated in the interpretation of the results and in the writing of the manuscript. All authors read and approved the final manuscript.

## Supplementary Material

Additional file 1 **Table S1. Sequencing parameters of the biological replicas of the studied genotypes.** Genotype symbols: Wild type (WT), Heterozygous (He), Homozygous (Ho). Letters a, b and c symbolize different experiments for each genotype. Technical replicates were grouped (ie: WT16ab). Click here for file

Additional file 2 **Table S2. Differential expression analysis.** baseMean (mean expression level, at the base scale, as a joint estimate from both conditions); baseMeanA (mean expression level, at the base scale, as an estimate for condition A); baseMeanB (mean expression level, at the base scale, as an estimate for condition B); foldChange (fold change from the first to the second condition); log2FoldChange (logarithm to basis 2 of the fold change); pvalue (significance of the fold change); padjusted (p values adjusted for multiple testing with the Benjamini-Hochberg procedure, which controls false discovery rate); resVarA (ratio of the single gene estimates for the base variance to the fitted value in condition A); resVarB (ratio of the single gene estimates for the base variance to the fitted value in condition B). Click here for file

Additional file 3 **Table S3. RPKM normalization of gene counts.** Genotype symbols: Wild type (WT), Heterozygous (He), Homozygous (Ho). Letters a, b and c symbolize different experiments for each genotype.Technical replicates for a same genotype sample were grouped (ie: WT16ab). Click here for file

Additional file 4 **Table S4. Number of genes significantly misregulated in homozygous or heterozygous *sall *mutant embryos in comparison to WT.** Genotype symbols: Wild type (WT), Heterozygous (He), Homozygous (Ho). Downregulated (D) or upregulated (U) genes in the second genotype with respect to the first one; E, no significant changes in expression between the two genotypes. Click here for file

Additional file 5 **Figure S1. Dosage effect of *sall *genes.** (A) mRNA-Seq expression plot for *arm, Fas-3, Nrg, Fas2, CadN and N* genes in heterozygous (He) and homozygous (Ho) *sall* mutant embryos at stage 16 showed consistent results with the differences in protein levels observed by Cantera et al. (Cantera et al. 2002). Notice that heterozygous *sall* mutant embryos have higher transcript levels than homozygous for all these adhesion and cytoskeleton genes, suggesting a dosage effect of Sall. (B) mRNA-Seq analysis of the transcriptome of WT16*,* He16 and Ho16 embryos, showed that five genes are differentially expressed (p < 0.01) between all the genotypes compared at stage 16 and have intermediate levels of expression in He16. (C) At stage 17, instead, four of the genes that are differentially expressed (p < 0.01) between all the genotypes compared at this stage had intermediate levels of expression in He17. Click here for file

Additional file 6 **Figure S2. Reads mapped along *IM3 *and *dro5 *genes.** (A, B) 36 or 38 long reads represented by grey lines map on *dro5* (A) or *IM3* (B) genes. Gene, mRNA and coding sequence (CDS) are represented on the upper part of each figure. On the left, the different biological replicates of each genotype analyzed are indicated. Click here for file

Additional file 7 **Figure S3. Putative Sall binding sites in regulated genes.** Graphical representation of *dro5* (A) and *IM3* (B) genes and the putative Sall binding sites (pink box in A, green and blue boxes in B) in the genomic region. Conservation of these sequences in various *Drosophila* species is depicted below the graphs. Click here for file

Additional file 8 **Table S5. Misregulated genes in homozygous *sall *mutant embryos.** Genotype symbols: Wild type (WT), Heterozygous (He), Homozygous (Ho). Downregulated (D) or upregulated (U) genes in the second genotype with respect to the first one; E, no significant changes in expression between the two genotypes. Genes previously associated with neurodegeneration/neuroprotection are indicated in RED. Click here for file

Additional file 9 **Unbiased Gene Ontology analysis of the genes significantly misregulated in homozygous *sall *mutant embryos using the VLAD online tool.** Genes misregulated in homozygous *sall* mutant embryos at stage 16, 17 and at the transition between both stages (genotype comparisons: WT16 vs Ho16; WT17 vs Ho17; Ho16 vs Ho17). Click here for file

Additional file 10 **Table S6. Functional classification of genes misregulated in homozygous *sall *mutant embryos with respect to wild type embryos at stage 16.** Genotype comparison WT16 vs Ho16. Wild type (WT), Homozygous (Ho).**Table S7. Functional classification of genes misregulated in homozygous *sall *mutant embryos with respect to heterozygous embryos at stage 16.** Genotype comparison Ho16 vs He16. Homozygous (Ho), Heterozygous (He). **Table S8. Functional classification of genes misregulated in homozygous *sall *mutant embryos with respect to wild type embryos at stage 17.** Genotype comparison WT17 vs Ho17. Wild type (WT), Homozygous (Ho). **Table S9. Functional classification of genes misregulated in homozygous *sall *mutant embryos with respect to heterozygous embryos at stage 17.** Genotype comparison Ho17 vs He17. Homozygous (Ho), Heterozygous (He). **Table S10. Functional classification of genes misregulated in homozygous *sall *mutant embryos at the transition from stage 16 to 17.** Genotype comparison Ho16 vs Ho17. Homozygous (Ho). **Table S11. Functional classification of genes misregulated in wild type embryos at the transition from stage 16 to 17.** Genotype comparison WT16 vs WT17. Wild type (WT). Click here for file

Additional file 11 **Figure S4. Functional classification of misregulated genes.** (A-C) Graphic representation of the main functional groups enriched in misregulated genes in the indicated genotypes, expressed as percentage of genes in each group. In red are marked the groups significantly overrepresented with respect to the total *Drosophila* genome with p < 0.01 and in green with p < 0.05. (A) Classification of the genes misregulated in Ho16 compared with He16. (B) Classification of the genes misregulated in Ho17 compared to He17. (C) Classification of the genes misregulated in WT embryos at the transition from stage 16 to 17. Click here for file

Additional file 12 **Table S12. Genes regulated by Sall both in embryonic stages and in and pupal muscle.** Click here for file

Additional file 13 **Table S13. Oligonucleotides used for PCR analysis.** Click here for file

Additional file 14 **Table S14. Functional Groups reference list.** *GO categories constructed from specific literature sources. Click here for file
