# Supplementary figures and images for: Whole transcriptome analysis of a reversible neurodegenerative process in Drosophila reveals potential neuroprotective genes
Source: BMC Genomics. 2012 Sep 15;13:483. doi: 10.1186/1471-2164-13-483 (PMC3496630; doi:10.1186/1471-2164-13-483)

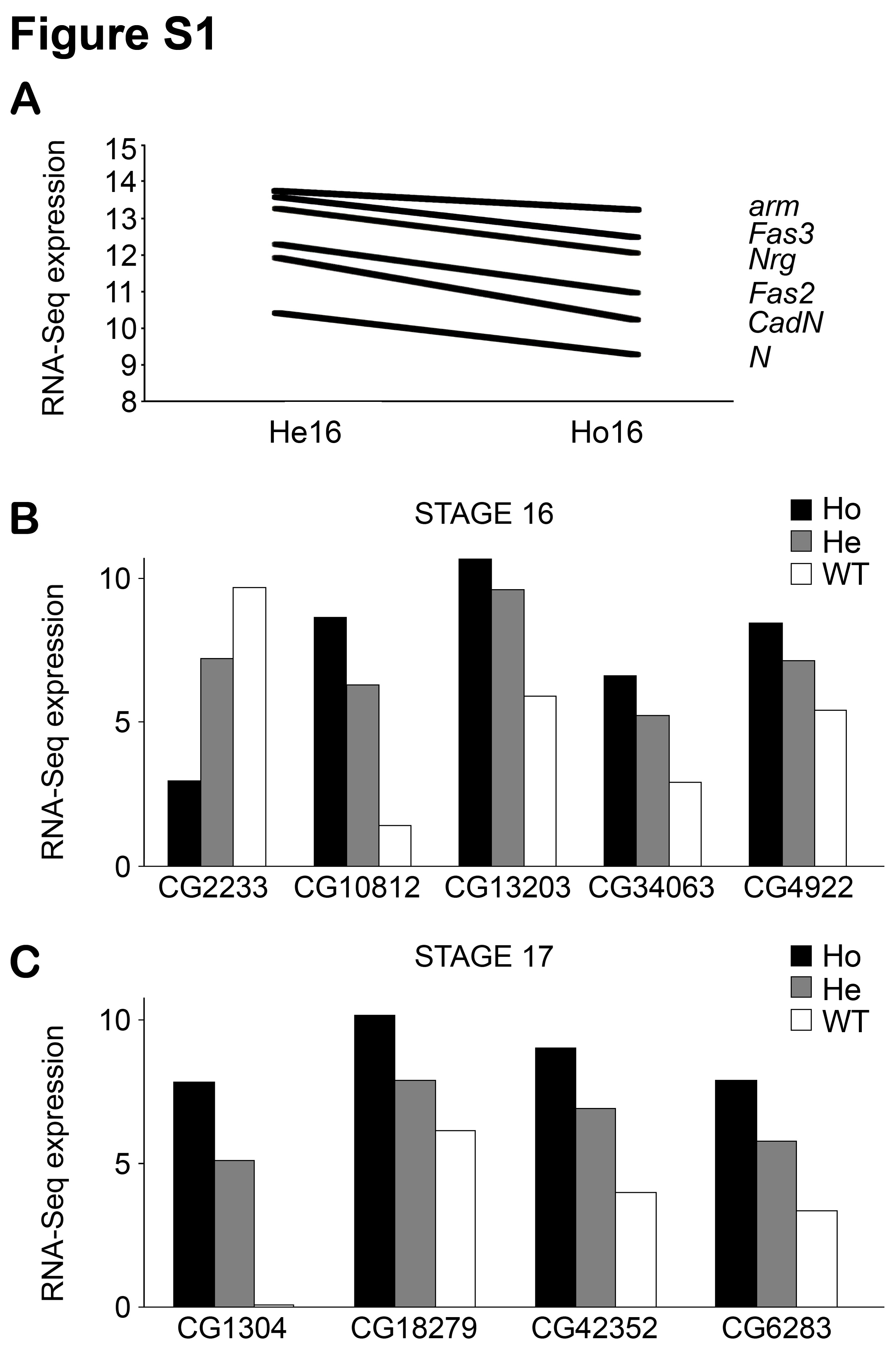

Supplement: Additional file 5 — Figure S1. Dosage effect of sall genes. (A) mRNA-Seq expression plot for arm, Fas-3, Nrg, Fas2, CadN and N genes in heterozygous (He) and homozygous (Ho) sall mutant embryos at stage 16 showed consistent results with the differences in protein levels observed by Cantera et al. (Cantera et al. 2002). Notice that heterozygous sall mutant embryos have higher transcript levels than homozygous for all these adhesion and cytoskeleton genes, suggesting a dosage effect of Sall. (B) mRNA-Seq analysis of the transcriptome of WT16, He16 and Ho16 embryos, showed that five genes are differentially expressed (p < 0.01) between all the genotypes compared at stage 16 and have intermediate levels of expression in He16. (C) At stage 17, instead, four of the genes that are differentially expressed (p < 0.01) between all the genotypes compared at this stage had intermediate levels of expression in He17. [file 1471-2164-13-483-S5.jpeg]

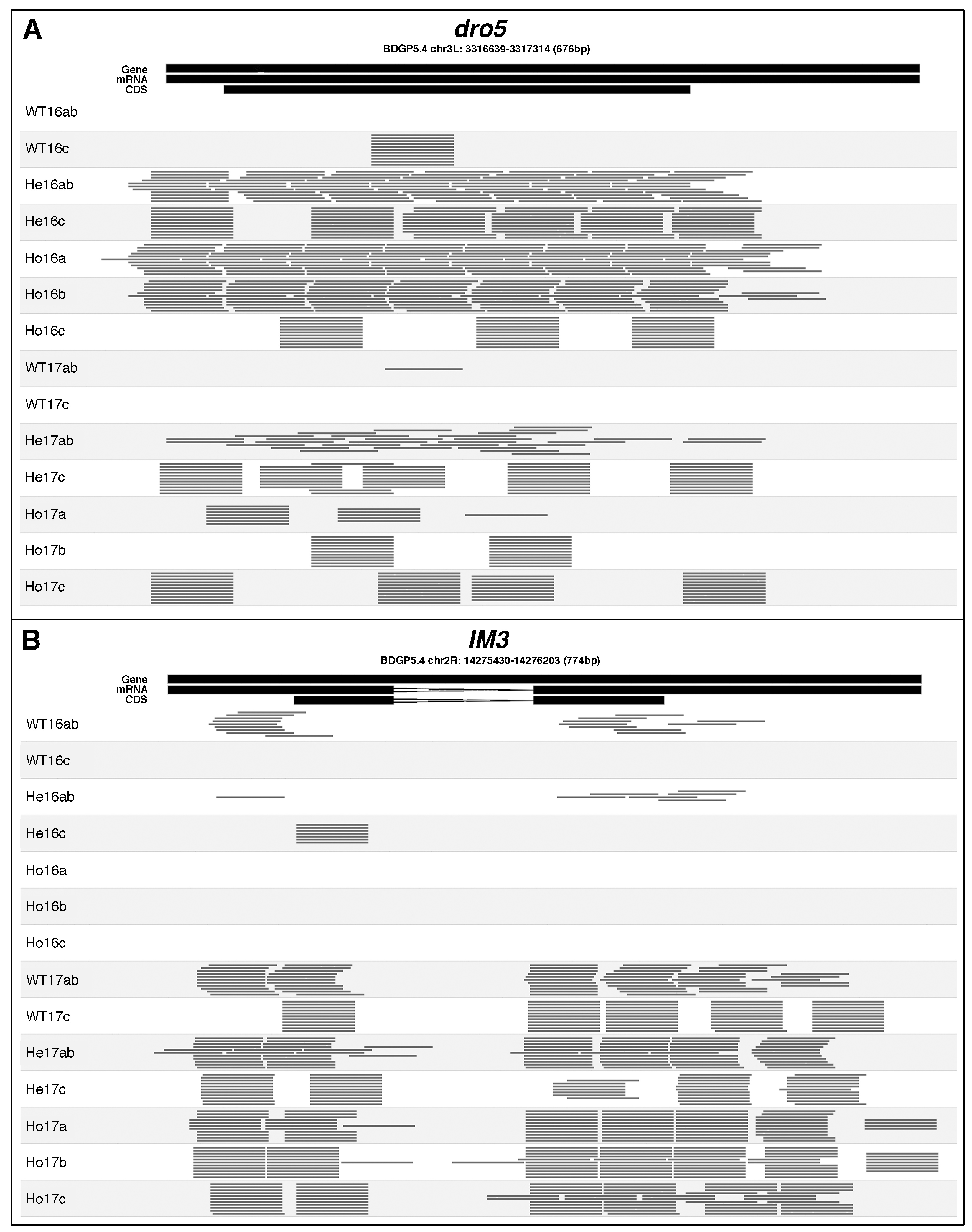

Supplement: Additional file 6 — Figure S2. Reads mapped along IM3 and dro5 genes. (A, B) 36 or 38 long reads represented by grey lines map on dro5 (A) or IM3 (B) genes. Gene, mRNA and coding sequence (CDS) are represented on the upper part of each figure. On the left, the different biological replicates of each genotype analyzed are indicated. [file 1471-2164-13-483-S6.jpeg]

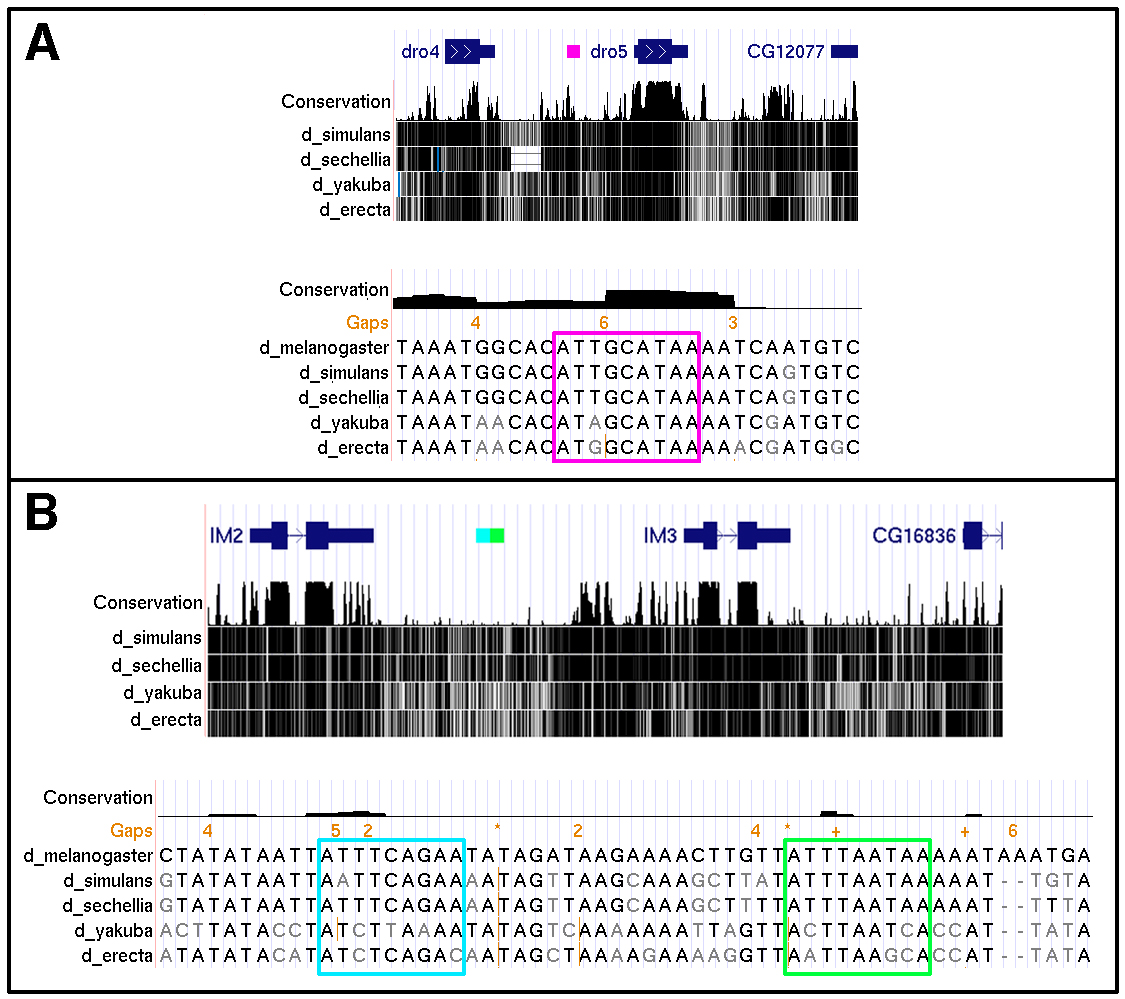

Supplement: Additional file 7 — Figure S3. Putative Sall binding sites in regulated genes. Graphical representation of dro5 (A) and IM3 (B) genes and the putative Sall binding sites (pink box in A, green and blue boxes in B) in the genomic region. Conservation of these sequences in various Drosophila species is depicted below the graphs. [file 1471-2164-13-483-S7.jpeg]

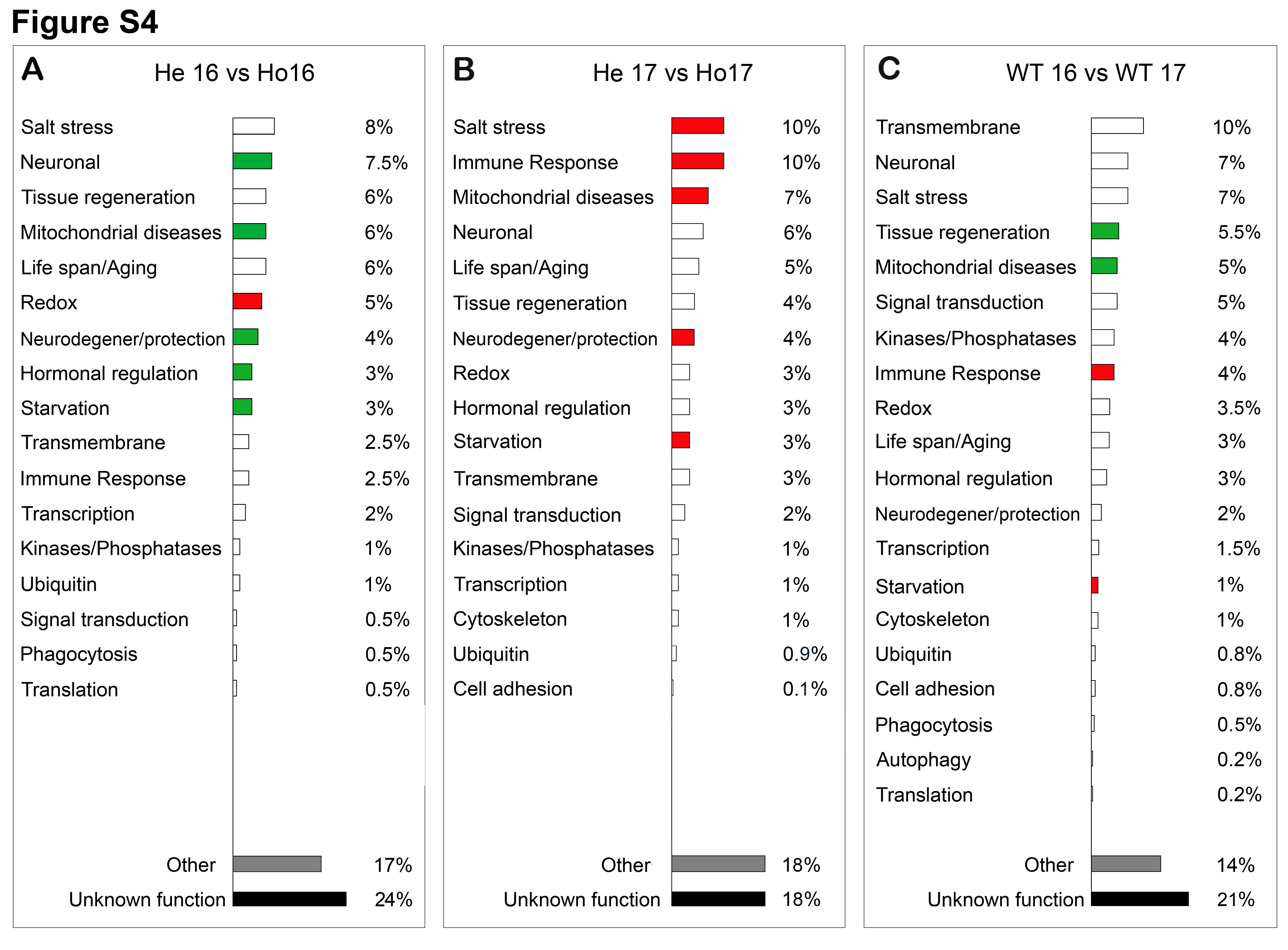

Supplement: Additional file 11 — Figure S4. Functional classification of misregulated genes. (A-C) Graphic representation of the main functional groups enriched in misregulated genes in the indicated genotypes, expressed as percentage of genes in each group. In red are marked the groups significantly overrepresented with respect to the total Drosophila genome with p < 0.01 and in green with p < 0.05. (A) Classification of the genes misregulated in Ho16 compared with He16. (B) Classification of the genes misregulated in Ho17 compared to He17. (C) Classification of the genes misregulated in WT embryos at the transition from stage 16 to 17. [file 1471-2164-13-483-S11.jpeg]
